# Tools Are Needed to Promote Sedation Practices for Mechanically Ventilated Patients

**DOI:** 10.3389/fmed.2021.744297

**Published:** 2021-11-12

**Authors:** Tao Wang, Dongxu Zhou, Zhongheng Zhang, Penglin Ma

**Affiliations:** ^1^Critical Care Medicine Department, Guiqian International General Hospital, Guiyang, China; ^2^Department of Emergency Medicine, Sir Run Run Shaw Hospital, Zhejiang University School of Medicine, Hangzhou, China

**Keywords:** mechanical ventilation, suboptimal sedation, patient tolerability, assessment tools, sedation depth

## Abstract

Suboptimal sedation practices continue to be frequent, although the updated guidelines for management of pain, agitation, and delirium in mechanically ventilated (MV) patients have been published for several years. Causes of low adherence to the recommended minimal sedation protocol are multifactorial. However, the barriers to translation of these protocols into standard care for MV patients have yet to be analyzed. In our view, it is necessary to develop fresh insights into the interaction between the patients' responses to nociceptive stimuli and individualized regulation of patients' tolerance when using analgesics and sedatives. By better understanding this interaction, development of novel tools to assess patient pain tolerance and to define and predict oversedation or delirium may promote better sedation practices in the future.

## Introduction

Mechanically ventilated (MV) patients can have a wide variety of discomforts resulting from multiple sources, including pathophysiological abnormalities (such as fever, hypoxia, and shock), emotional alterations (anxiety or fear), and intensive care procedures as well (such as non-physical ventilation, immobilization, frequent puncturing, and turning over, etc.) (1–4). Analgesics and sedatives are often used to maintain MV patients' comfort (5). In the last two decades, it has been observed that MV patients were deeply sedated very frequently in intensive care units (ICU) (6–8). Significantly, this behavior has been associated with poor outcomes, including prolonged duration of mechanical ventilation, increased incidence of ventilator associated pneumonia decline in cognitive ability, and even increased long-term mortality (6–10). Therefore, it has been strongly recommended to optimize sedation practices, such as implementing a light sedation protocol and the eCASH concept (early Comfort using Analgesia, minimal Sedatives and maximal Humane care) in MV patients (11, 12). Recently, more days without occurrence of coma or delirium were demonstrated in the patients receiving no sedation protocol than in those who were maintained at even light levels of sedation during the stay in the ICUs (13). These findings indicated that the lighter the level of sedation, the better outcomes would be for MV patients.

However, the frequency of deep sedation remains high in clinical practice based on recently published data from various studies (13–15), although a strong recommendation of minimizing sedation for MV patients has been published in the updated guidelines for several years. For instance, the mean depth of sedation was below RASS−2 (mean RASS = −2.3) on day 1 in the sedation group (i.e., the usual care arm) of Olsen's randomized control trial in ICUs where no sedation strategy was initiated 10 years ago (13, 16). It was previously recognized that the low adherence to a minimal sedation protocol was multifactorial, including inadequate assessments because of shortage of nurses, lack of multidisciplinary cooperation, and even misperception as well (17–20). However, the barriers to translating a minimal sedation protocol into standard care for MV patients are not well-defined. It is necessary to reveal fresh insight into the fact that the outcome favored minimal sedation protocol was poorly implemented in MV patients.

## Lightly Sedated Patients' Tolerance to Mechanical Ventilation

Lack of knowledge on patient intolerance to MV has been an important barrier to implementing a minimal sedation protocol in MV patients. Among the common signs of patient intolerance, agitation affected nearly half of ICU MV patients in previous reports (21, 22). Moreover, accumulating evidence has demonstrated that the risks of agitation or agitation-associated events were significantly increased while maintaining MV patients at light levels of sedation (usually defined as at levels of RASS from −2 to 1) (16, 23, 24). Notably, severe agitation has been associated with unplanned self-extubation, removal of important intralumenal tubes and vascular catheters, poor patient–ventilator synchrony, and increased morbidity, including PTSD (25–28). Accordingly, agitation or agitated adverse events have been of serious concern in most ICU nurses and physicians, which in turn has affected their willingness to implement light sedation practices in their routine clinical care (18, 29). In a nationwide cross-sectional survey, we also found that ICU physicians' perception of patients' tolerance to the support levels of ventilation with light sedation was highly varied across institutions. Importantly, their perceptions were largely translated into clinical practices (14). In addition, bolus administration of sedatives was usually given as a rescue intervention for agitation, which often led to unjustified deep sedation (18).

Actually, measurement of MV patients' tolerance (who are unable to communicate) remains problematic. Tools to evaluate patient tolerance or sedation depth in mechanical ventilation have evolved since the Ramsay sedation scale first used in 1974 as shown on Table 1. RASS offers broader discrimination in the mild-to-moderate sedation range. It is the most commonly used tool in clinical practice (41), and has demonstrated greater inter-rater reliability between clinical staff (37, 38, 42, 44). Therefore, frequent assessment of RASS has been strongly recommended to optimize the depth of sedation for MV patients and has been associated with improvement in outcomes (56). However, RASS, like other tools, is actually a transient result of patient tolerance to nociceptive stimuli as regulated by the infused analgesics and sedatives in MV. It is not a scale to directly assess the intensity of stimuli that patients experience instantaneously. Being complementary to RASS, the pain assessment tools such as Behavioral Pain Score (BPS) or Clinical Pain Observation Tool (CPOT) were suggested to improve the overall assessment of comfort of critically ill patients. However, the intensity of nociceptive stimuli might change over time because of occurrence of fever, thirst, drainage tube pain, or intestinal colic, etc., that would raise the risk of patient intolerance to MV (or vice versa). No matter how frequent the RASS assessment is, titration of analgesics and sedatives always lags behind patient intolerance (or oversedation), which partially at least accounts for frequent and unpredictable agitation. In fact, there is a lack of reliable criteria to scale responses to the stimuli that patients experience during MV. Accordingly, it is difficult for ICU physicians to properly estimate the intensity of patients' responses as well as their tolerance when patients are lightly sedated, which might be an important source of suboptimal sedation practices.

**Table 1:** Tools for assessment of sedation depth or pain in mechanically ventilated patients.

**Tools**	**Describe**	**Advantage**	**Disadvantage**	**Clinical studies**	
				**Comparator**	**Findings**
RSS Dawson et al. (30)	The RSS is a single-item tool to measure consciousness across three levels in critically ill patients who are awake and three levels in patients who are judged to be asleep (31).	The earliest and the most widely used scale (32).	Use of a single item to assess two or more different aspects of sedation can lead to loss of clinically important information and systematic or random measurement error (33).	SAS (33)	No difference was found in validity between two scales
NICS Mirski et al. (34)	The NICS is a simpler, more intuitive sedation scale that is both easy to use and recall and favored by nurses as a sedation communication tool (34).	NICS ranked highest in nursing preference and ease of communication and may thus permit more effective and interactive management of sedation (34).	Subjective.	• RASS • RSS • SAS	NICS is a valid and reliable sedation scale for use in a mixed population of intensive care unit patients (34).
SAS Mirski et al. (34)	The SAS is a single-item seven point scale developed by Riker and colleagues and commonly used within ICU (35).	Both reliable and valid (36)	Not suitable for patients with hearing impairment, nerve damage, and hemiplegia (37)	RSS (33, 38)	• The SAS provides additional information by stratifying agitation into three categories without sacrificing validity or reliability (33). • The SAS showed the best correlation and the best agreement results in all professional categories (38).
ATIC De Jonghe et al. (39)	The ATICE consists of five items: Awakeness and Comprehension combined in a Consciousness domain, and Calmness, Ventilator Synchrony, and Face Relaxation combined in a Tolerance domain (39).	Evaluates sedation and tolerance; longitudinal validity demonstrated; explicit instructions provided (39).	Studied in medical patients; only properties may differ in surgical population; more complex scoring method-requires (39).	• RASS • RSS • SAS	Offers assessment of tolerance to the ICU environment;longitudinal validity demonstrated (39).
RASS Sessler et al. (40)	The RASS is a single-item scale that has 10 levels of response, which range from minus five to plus four.	Longitudinal validity demonstrated in diverse patient. It offers broader discrimination in the mild-to-moderate sedation range (41, 42).	If there are visual or auditory obstacles, it will affect the accuracy of the evaluation results (43) physical stimulation can increase anxiety of patient.	• RSS (34, 38, 41, 44, 45) • SAS (34, 37, 41) • MAAS (34)	• The RASS correlated more highly with BIS compared to RSS (39), and demonstrated greater inter-rater reliability between clinical staff compared to RSS and SAS (37, 38, 42, 44). • The RASS showed high levels of reliance and ease of use in scoring and communicating sedation, agitation and intuitiveness, compared to the RSS, MAAS, and SAS (34).
BIS Watson and Kane-Gill (46)	The BIS measures the level of sedation by integrating information from the electroencephalography and a mathematical technique referred to as bispectral analysis (46).	Offers objective monitoring; offers continuous monitoring; Continuous monitoring (47)	Variability; conflicting ICU validity results; muscle activity alters values; Unable to distinguish between natural sleep and drug-induced sleep (48).	• RASS • RSS • SAS • ATICE • MAAS	The BIS monitor has potential benefits in the ICU environment, although optimal use requires further investigation (46).
MAAS Devlin et al. (49)	The MAAS is also a single-item tool with seven response-defined categories of behavior, which originated from the SAS and is therefore structurally similar to the SAS (49).	The MAAS was superior to the LSS based upon the observation that MAAS scores were less variable (50).	There is insufficient evidence to warrant use of the MAAS as a new method of evaluating critically ill patients requiring sedation in the emergency department (50).	LSS (50)	The MAAS is a valid and reliable sedation scale for use with mechanically ventilated patients in the SICU (49).
BPS Payen et al. (51)	The BPS was based on a sum score of three items: facial expression, movements of upper limbs, and compliance with mechanical ventilation (51).	• Higher reliability shown for the muscular domain (52). Simplicity, easiness; Descriptors clear or precise (53).	• Discriminant validation seems less satisfactory in sedated or agitated patients (52). • Less specific (53).	CPOT	Both CPOT and the BPS showed good reliability and validity and were good options for assessing pain during painful procedures with intensive care unit patients unable to self-report on pain (54).
CPOT Gélinas et al. (55)	The CPOT scale includes four behavioral indicators:facial expression;body movements;muscle tension; and compliance with the ventilator (for intubated patients) or verbalization (for extubated patients) (55).	• Have particularly good reliability and validity in assessing pain during procedures (54). • Descriptors more detailed; Descriptors better described (53).	• Discriminant validation seems less satisfactory in sedated or agitated patients (52). • Descriptors less well-detailed or confusing (53).	BPS	Because of the discriminant validation, the CPOT is to be preferred (54).

*SAS, Ramsay Sedation Scale; SAS, Riker Sedation-Agitation Scale; RASS, Richmond Agitation Sedation Scale; AITCE, Adaptation to the Intensive Care Environment; NICS, Nursing instrument for the communication of sedation; BIS, Bispectral index; LSS, Luer Sedation Scale; BPS, Behavioral Pain Scale*.

Burk et al. (26) previously reported several predictors of agitation within 24 h in adult critically ill patients, including Sequential Organ Failure Assessment score, PaO_2_/FiO_2_ <200 mmHg, receiving MV, using restraints, etc. Based on the variables relating to fever, ventilator settings, alterations in respiratory physiology, and dosage of sedatives and analgesics, our study group recently developed an ensemble model for the prediction of agitation in invasive MV patients under light sedation (57). The model showed good calibration and discrimination in an independent dataset. However, the effectiveness of interventions based on the prediction model need to be investigated in further experimental trials. These findings indicate that agitation (i.e., severe patient intolerance in MV) is predictable by evaluating variables related to nocioceptive stimuli. Thus, development of a tool for evaluating the balance between the intensity of stimuli and patient tolerance when analgesics and sedatives are used is needed to implement a minimal sedation protocol in the future.

## Recognition, Estimation, and Prevention of Oversedation in MV Patients

Suboptimal sedation practices include both oversedation and undersedation. In the literature, numerous studies have shown that deep sedation continues to be common in the ICU (8, 9, 13–15). Generally, it has been recognized that deep sedation (below RASS−2) remains relevant only for the management of some situations in MV patients, such as severe acute respiratory distress syndrome with ventilator–patient asynchrony or with use of neuromuscular blocking agents, severe brain injury with intracranial hypertension, status epilepticus, etc. (58–61). For the vast majority of ICU MV patients, deep sedation is unnecessary and should be avoided (62). Oversedation is therefore suspected when MV patients are sedated at the depths below RASS−2. However, this concept is mainly based on expert opinions rather than empirical evidence, which is misleading for appropriate sedation practices. For instance, sedatives could be overused while maintaining the level of sedation at RASS−2 for MV patients ready for weaning. On the other hand, the sedation depth at RASS−3 (or even the deeper levels) might be necessary for acute critically ill patients with multiple organ dysfunction caused by aggressive inflammatory responses (63, 64). In fact, no consensus on the definitions of deep sedation and oversedation is available because of gaps in the evidence. There is a dearth of information regarding the interaction among sedative choice, sedation depth, and patient-specific factors that affect outcomes (65). Therefore, determining optimal sedation and oversedation in MV patients remains challenging.

Ambiguity in definition is an important barrier to the development of protocols to prevent oversedation in practice. Previously, the ABCDEF bundle (Assess, prevent, and manage pain; Both spontaneous awakening and breathing trials; Choice of analgesia and sedation; Delirium assess, prevent, and manage; Early mobility and exercise; Family engagement/empowerment) was developed to promote appropriate sedation practices by creating a safe and comfortable environment for MV patients (66). Although reduction in the rate of deep sedation and improvement in outcomes were demonstrated in patients who did receive more of the bundle elements each day, the major limitation was low adherence in clinical practice because of too many unresolved issues involved in this protocol (67). A novel sedation-monitoring technology (the Responsiveness Index, RI) based on facial electromyography was developed to provide an alert for possible deep sedation. Results showed that use of the monitor increased optimal sedation-analgesia quality but just by 7% (68). Results from the AWARE study (69) revealed that by decreasing use of intravenous hypnotics, the oversedation prevention protocol was feasible in clinical practice and resulted in a significantly earlier time to spontaneous breathing trial and reduced duration of mechanical ventilation (69). However, mortality was not significantly different between the study group and the control group. It should be interpreted with caution that the rate of oversedation or deep sedation was prevented in this study. Therefore, a precision definition is fundamental for development of a reliable scale for estimation as well as an effective protocol for prevention of oversedation in MV patients.

## Delirium Prediction

Delirium is a well-established syndrome in the ICU that is considered to be an acute onset of brain dysfunction (70). There are two motor subtypes of delirium that are categorized according to its clinical presentation, namely, the hyperactive and hypoactive subtypes (71, 72). The primary presentation of hyperactive delirium is agitation, which is reported to occur in many ICU patients (26). Although agitated delirium is found to be less harmful than the hypoactive type with respect to 12-month mortality (72, 73), potential serious consequences of agitation as opposed to its hypoactive counterpart, mentioned above included medical device removal (such as urinary catheter, venous or arterial line, or surgical drain), falling out of bed, immobilization device removal, or self-aggression or aggression toward medical staff (25–28, 74). Thus, the prediction and appropriate prevention of agitated delirium is of paramount importance in the management of MV patients.

The mechanism of delirium remains unclear (75). Risk factors for delirium include illness-related acute pathophysiological abnormalities (e.g., hypotension, acidosis, hypoxia, and sepsis), environmental factors (e.g., lighting, alarm sounds, and noise); and iatrogenic harm (e.g., frequent suctions, puncture, immobilization, and even use of analgesic and sedative drugs) (76–79). Among these, there are potentially modifiable risk factors, for example, minimizing sedation and benzodiazepine use (80). Significantly, numerous studies have reported that patients receiving deep sedation were more susceptible to post-traumatic stress disorder syndrome, ICU memory disorder, and delirium (81, 82). On the other hand, two recently published meta-analyses revealed that delirium occurred more frequently in the light than in the deep sedation group of MV patients (24, 83). Because of multiple etiologies, therefore, prediction and prevention of delirium remains problematic.

Some prediction models have been developed for delirium, but limitations remain. For example, the prediction model for delirium (PRE-DELIRIC) and early prediction model for delirium (E-PRE-DELIRIC) were initially developed in a single hospital and validated in four hospitals (84). However, the discriminatory ability of these models in an external dataset was less than satisfactory (area under curve: 0.68–0.79, respectively) (85–87). These studies are limited in several aspects. First, previous studies typically used variables collected on the day of ICU admission, and the delirium event may happen several days later. Some physiological variables change significantly in this interval. Second, there is no model to specifically predict hypoactive delirium. Third, previous models were usually developed in a single center, which partly explains the models' suboptimal performance in an external dataset. Foruth, the previous models were developed as generalized linear models that failed to capture higher- order and interaction terms between predictors. Therefore, a novel delirium prediction model is needed for MV patients.

## Conclusion

Suboptimal sedation practices are common, which are largely attributable to the evidence gaps concerning the intensity of nociceptive stimuli that patients experience and patients' tolerance and its treatment by using analgesics and sedatives. Development of novel tools to assess patient tolerance and to define and predict oversedation or delirium are needed to implement better sedation practices in the future.

## Data Availability Statement

The original contributions presented in the study are included in the article/supplementary material, further inquiries can be directed to the corresponding author/s.

## Author Contributions

TW and PM were the major contributors in writing the manuscript. DZ and ZZ helped to revise the manuscript. PM critically reviewed the manuscript and agreed with the final version. All authors read and approved the submitted manuscript.

## Conflict of Interest

The authors declare that the research was conducted in the absence of any commercial or financial relationships that could be construed as a potential conflict of interest.

## Publisher's Note

All claims expressed in this article are solely those of the authors and do not necessarily represent those of their affiliated organizations, or those of the publisher, the editors and the reviewers. Any product that may be evaluated in this article, or claim that may be made by its manufacturer, is not guaranteed or endorsed by the publisher.
